# Effects of Atrazine on the Development of Neural System of Zebrafish, *Danio rerio*

**DOI:** 10.1155/2015/976068

**Published:** 2015-05-31

**Authors:** Hao Wang, Shumei Mu, Fengjuan Zhang, Hailing Wang, Huan Liu, Han Zhang, Xianjiang Kang

**Affiliations:** College of Life Sciences, Hebei University, Wusi East Road 180, Baoding 071002, China

## Abstract

By comparative analysis of histomorphology and AChE activity, the changes of physiological and biochemical parameters were determined in zebrafish embryos and larvae dealt with atrazine (ATR) at different concentrations (0.0001, 0.001, 0.01, 0.1, and 1 mg/L). This study showed that the development of the sarcomere and the arrangement of white muscle myofibers were affected by ATR significantly and the length of sarcomere shortened. Further analysis of the results showed that the AChE activity in juvenile fish which was treated with ATR was downregulated, which can indicate that the innervation efficiency to the muscle was impaired. Conversely, the AChE activity in zebrafish embryos which was treated with ATR was upregulated. A parallel phenomenon showed that embryonic primary sensory neurons (Rohon-Beard cells), principally expressing AChE in embryos, survived the physiological apoptosis. These phenomena demonstrated that the motor integration ability of the zebrafish was damaged by ATR which can disturb the development of sensory neurons and sarcomere and the innervations of muscle.

## 1. Introduction

Atrazine (2-chloro-4-ethylamino-6-isopropylamine-s-triazine, ATR) is a widely used triazine herbicide to control broadleaf weeds and some gramineous grasses selectively. ATR blocks the process of electron transport in photosystem II in target plants via competition with plastoquinone Q_B_ at its binding site. Three possible detoxification pathways for ATR exist in higher plants: (1) the dealkylation of cytochrome P450 in photosystem I, (2) the covalent modification of glutathione transferase (GSTs) in photosystem II, and (3) the hydrolysis of aromatic ring with nonenzymatic catalysis [1]. ATR causes a long term of soil and water pollution by spraying farmland, precipitation, and so forth.

The toxic effects of ATR on animals are embodied in the deformity of reproductive organ, carcinogenicity, hypoevolutism, hypoimmunity, and so forth [2, 3]. ATR and its metabolites known as environmental endocrine disruptors (EDCs) have the neuroendocrine toxicity [4]. ATR interferes in endocrine balance by acting on the central nervous system [5–7]. It has been proved that ATR and its metabolites affect the central nervous system through controlling the hypothalamic-pituitary-gonadal axis (HPG) and then affect the development of the reproductive system. Recently, many researches focused on how ATR disrupts the function of thalamus neuroendocrine system. The undifferentiated neuronal precursor cells or living brain tissue exposed with ATR* in vitro* and the levels of catecholamine neurotransmitters (dopamine and norepinephrine) synthesized from the neurons can be altered significantly [8]. The decline of norepinephrine in hypothalamus would inhibit the periodic releasing of gonadotropin releasing hormone (GnRH), while dopamine decline would lead to a decrease in the secretion of prolactin [9, 10]. It was suggested that catecholamine neurons in the central nervous system might be potential targets of ATR and its metabolites. Many researches also showed that ATR is an environmental factor which can cause neurodegeneration [11]. But the toxicological effects of ATR on central nervous were not researched sufficiently.

The development process of zebrafish (*Danio rerio*) is rapid. The neural tube forms in 24 hours postfertilization (hpf) and then generates tactile sense within 27 hpf. The nerve reflex system develops in a short time. Therefore, zebrafish are exceptionally well suited for developmental neurotoxicity studies [12–14].

The applications of zebrafish model to monitor potential neurotoxic effects have been studied in recent researches. Zebrafish embryos after short-term exposure to ethanol at low concentration show that the developmental defects produced by ethanol are similar to the human fetal alcohol syndrome (FAS), such as the decrease of eye diameter, the deformity of central nervous system and bones, the decline in learning and memory, the dent of the ability of escape, and so forth [15, 16]. Parkinson's disease (PD) is a kind of nervous system degenerative diseases caused by the loss of dopaminergic neurons. Many environmental contaminants, such as MPTP (1-methyl-4-phenyl-5-tetrahydropyridine), rotenone, and paraquat, are proposed to have relevance to inducing PD. These toxicants lead to oxidative stress, energy metabolic disorders, and protease inactivation by inhibiting the mitochondrial redox enzyme system and then cause the death of dopaminergic neuron. Zebrafish larvae exposed to such substances showed bradykinesia and reducing of dopaminergic neurons [11].

In this study, zebrafish was used as model to study the cellular mechanism of the toxicity effect of ATR on central nervous system and the innervations of organism movements during the embryonic development. It focused on the toxicity effect of ATR on the development and function of nervous system in vertebrate. This study used the means of conventional cytobiology morphological observation and biochemical detection to reveal the critical period and important part sensitive to the external environment factors in the actual development process of vertebrate and provided an effective method to the assessment of the neurotoxicity of environmental pollutants.

## 2. Material and Methods

### 2.1. Zebrafish and Embryos

Adult wild zebrafish (over 5-month-old) were purchased from an aquarium market (Baoding, Hebei, China). Males and females were housed separately in glass tanks with 20 cm depth aerated water at 25 ± 3°C. The stocking density was 5–8 fish/L water. Fish were kept on a 14 h light : 10 h dark photoperiod and fed three times a day.

The day before spawning, females and males were placed in the same tank at the proportion of 1 : 2 or 1 : 3 (female : male) and separated by vertical sieve. The next day at dawn, when removing partitions, the parent fish began to mate with light. After 30 min the parent fish were removed and fertilized eggs were collected synchronously. Embryonic age was calculated at this time. The collected fertilized eggs were washed with 40 mg/L sea salt solution (25°C) 2-3 times.

### 2.2. Exposure Experiments

ATR of ≥97.0% chemical purity (Shandong Jinan Kesai Agricultural Chemical Co., China) was dissolved in 10% Hank's solution with the solvent dimethyl sulfoxide (DMSO). The working concentration of DMSO was 0.1‰ (v/v) in each test group and the control.

At 2 hpf, the synchronous development embryos were selected and randomly distributed into six dishes. Five groups were exposed to a range of ATR concentrations of 0.0001, 0.001, 0.01, 0.1, and 1 mg/L, respectively; one group as a solvent control was dealt with DMSO. All the groups were repeated twice.

The density of culture was five embryos/mL. All the embryos were raised till 9 days postfertilization (dpf) at 28 ± 0.5°C, 14 h light : 10 h dark cycles. Half of the solution was changed a day.

### 2.3. Muscle Fiber Arrangement and Sarcomere Development

Since muscle fiber had anisotropy, the arrangement of muscle fibers in the trunk sarcomere of juvenile zebrafish was sought clearly with polarization microscope.

At 2 dpf, 4 dpf, and 6 dpf, five juveniles were picked up with a long glass dropper carefully and moved onto the slides. Culture medium was replaced with 90% glycerol to limit the movement of juvenile trunk.

The alignment direction and organization of muscle fibers in the juvenile fish at the three developmental stages were recorded during morphological observation. The proportions of slow twitch and fast twitch were recorded as the reference parameters after the fibers differentiation. The microscopic measurement about the sarcomere length of 6 dpf juvenile was taken. The measurement parameters included the length of sarcomere along the anterior-posterior axis and the height along the dorsal-ventral axis. The arithmetic mean of five sarcomeres from 16th to 20th was calculated as the single sarcomere length. The height of sarcomere was the distant of the dorsal and ventral sides in the single sarcomere fiber. The heights of the 16th sarcomere, 18th sarcomere, and 20th sarcomere were measured, respectively; the arithmetic average of the three measurements was calculated as a single sarcomere height. The experiments were repeated 6 batches.

### 2.4. Acridine Orange (AO) Stains the Whole Embryo to Reveal the Apoptotic Cells

Apoptosis* in vivo* distribution can be seen in live zebrafish embryos by incubating embryos with AO.

20 hpf zebrafish embryos were used in the study. With anatomical microscope, we carefully peeled egg membrane and avoided any extra extrusion. The embryos without egg membrane were immersed in 10% Hank's solution containing AO (2 *μ*g/mL) for 15 min at 28°C. Then removed the staining solution, and rinsed the stained embryos with 10% Hank's solution for 5 times, each time for 10 min. The embryos were placed into 96-well plates (10 embryos in each, 2 parallel reactions for each) and blotted up the culture fluid with absorbent papers. Each hole dropped 50 *μ*L precooled acetone for 10 min at −20°C then added 50 *μ*L ddH_2_O each hole for 10 min at room temperature to lyse cell. Plates were read at 492 nm (OD_492_) with an ELISA reader. Blank samples were prepared using unstained embryos operating in parallel. The difference between the OD of the stained and blank samples was the sample absorbance at 492 nm. When the difference ≥10%, the corresponding experimental groups were examined by light microscopy.

The embryos were examined by light microscopy dyed and rinsed as the above method and then the embryos were moved on a slide and placed on ice for 5 min to reduce embryonic tail swing. With the fluorescence microscope, we observed the distribution of dorsal spinal cord cells pigmented by AO.

### 2.5. Observing Rohon-Beard Neurons (RB Neurons) of the Embryo* In Vivo*

RB neurons have a big size, which were arranged in two rows along the both sides of anterior-posterior axis in live embryos. They can be clearly observed under the phase contrast microscope (PCM). RB cells appeared in 14 hpf and reached their peak at 30–48 hpf, reducing sharply at 60–72 hpf [17]. So the most obvious apoptosis appeared at 72 hpf.

The juveniles at 72 hpf were placed on the slides and then they were covered with coverslips as the samples observed by the PCM. The number of RB neurons was calculated under the PCM. The experiments were repeated 5 batches.

### 2.6. The Activity of Acetyl Cholinesterase (AChE)

Thirty embryos (larvae) were collected at 24 hpf, 36 hpf, 48 hpf, and 72 hpf, respectively, and then the embryos were homogenated with 100 *μ*L precooling fresh buffer (100 mM Tris, Cl pH 7.5, 50 mM NaCl, 30 mM KCl, 1% Triton X-100, 2 mM EDTA pH 8.0, 2 mM PMSF). The homogenate was centrifuged at 4°C (800 g × 10 min); the supernatant was collected as the tissue samples.

Coomassie brilliant blue were used to determine the sample protein concentration. AChE activity was determined by AChE kits (Nanjing Institute of Biological Engineering, China), operating according to its instruction, as follows: 50 *μ*L substrate solution and 50 *μ*L chromogenic liquid were added to 3 *μ*L samples, incubated 6 min at 37°C after blending, and then 3 *μ*L stop solution was added quickly to terminate reaction. 10 *μ*L transparent agent and 5 *μ*L stabilizer were added to mixed solution and placed at room temperature for 15 min. As the control, 3 *μ*L samples were added after termination reaction. Samples were instead of 1 mM AChE standard and ddH_2_O in the standard control and blank control, respectively. After the reaction, 100 *μ*L solution of each sample was removed to 96-well plates and then read at 405 nm (OD_405_) with an ELISA reader with double wavelengths mode.

The AChE activity unit (U) was defined that 1 *μ*mol AChE substrate was hydrolyzed by per mg protein at 37°C for 6 minutes. The AChE activities of tissues were determined according to formula (1):

(1) AChE  activity  U=ODsample−ODcontrolODstandard−ODblank×1total  protein  concentration  of  the  sample.

All samples used for the determination of protein content and the activity of AChE must be fresh, and the detect experiments should be done within 2 hours.

### 2.7. Statistical Analysis

The results were analyzed by SPSS 11.5 software; the Origin 7.0 mapping software was used for data mapping and trend-line fitting. All the statistical results of the experimental data were showed as the average ± standard error (*n* = valid sample cases). Experimental data of sampling number more than 10 were compared to normality test data in each group. One-way ANOVA was used for analysis the significance of difference between the test and control groups. The probability level used for the statistical significance was *P* < 0.05 (☆), *P* < 0.01 (☆☆), *P* < 0.005 (☆☆☆).

## 3. Results

### 3.1. Differentiation and Arrangement of Muscle Fibers inside of the Sarcomere Changed in the ATR Treatment

The development of zebrafish trunk sarcomere included the increasing number and differentiation of muscle fibers. The muscle fibers can be divided into fast twitch and slow twitch. Fast twitch fibers also called white muscle [18] are the main component of the sarcomere. Fast twitch has a continuous distribution in deep layer of the sarcomere and is arranged in parallel to the body axis. Fast twitch fibers can synchronize contraction and participate in C-shaped escape and glide swimming. Because of the rich myoglobin, the slow twitch fibers are also called red muscle. The slow twitch is paucity in zebrafish and is distributed in superficial layer of the sarcomere discontinuously. Slow twitch fibers are arranged at an angle with the body axis and participate in the continuous adjustment of the swimming of the trunk.

**Figure 1:**
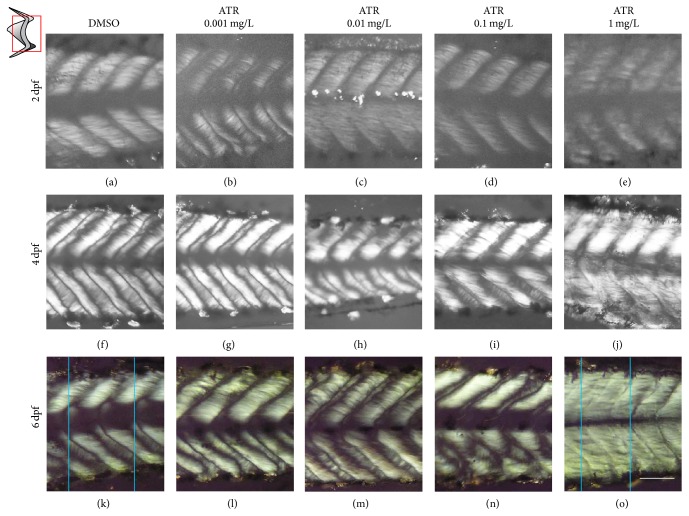
ATR disrupted the difference of axial muscle and the arrangement of muscle fiber of larvae treated at early embryonic stages. After hatching, the development of juveniles' sarcomere was observed in polarizing microscope photographs. At 2 dpf (a–e), the density of muscle fibers of all the groups including the control (a, DMSO) was low. Except for the group exposed to 0.001 mg ATR/L (b), there was no obvious differentiation of the fast and slow twitch. At 4 dpf (f–j), in the control group, the sarcomere developed normally, and the density of muscle fibers increased, but the differentiation of the fast and slow twitch was not significant (f); with the increase of concentration of ATR, the boundaries of muscle tended to be blurred (g–j, gradient ATR). The shape of sarcomere appeared to emerge at 6 dpf (k–o). In control group, the length (between the two vertical lines) of single sarcomere increased; muscle fibers were arranged stably (k); the organization pattern of ventral muscle fibers changed with the concentration of ATR (l–o, gradient ATR). The diagram at left upper corner was for the structure of sarcomere of bony fish trunk (gray for slow twitch in shallow sarcomere and white for fast twitch in deep sarcomere); the red box was for the photo display. In all photos, the dorsal of juvenile was up; the mouth was on the right side. Scale: 40 *μ*m. 5 larvae were used for each experiment group (2 dpf, 4 dpf, and 6 dpf), repeated 6 batches.

The changes of sarcomere development process in ATR treatment were observed in the experiment at first. Results showed that the density of muscle fibers in 2 dpf juvenile was low under normal conditions. The major component was the fast twitch fibers which were arranged in parallel and it had no obvious differentiation between fast and slow twitch muscles (Figure 1(a)). The content of muscle fiber increased significantly at 4 dpf. It showed that the muscle fibers were brighter than the normal; apparent differentiation with clear boundaries was also found in sarcomere. The proportion of fast twitch muscle was higher than the slow twitch muscle's in dorsal sarcomere and the number of fast twitch muscles was equal to the number of the slow twitch muscles in ventral sarcomere (Figure 1(f)). The 6 dpf juvenile sarcomere muscle reached into the stereotypes phase when the differentiation of the muscle and muscle tissue model had been fixed. The number of fast twitch muscles was more than slow twitch muscles in dorsal sarcomere and so was the trend in ventral sarcomere. The stretch of the sarcomere changed along with the increasing of the body length. Low concentration of ATR (0.001 mg/L) seemed to accelerate the development process of juvenile sarcomere; the differentiation of muscle fibers appeared at 2 dpf. Although the number was low, the arrangement was very similar to the normal juvenile's at 4 dpf (Figure 1(b)). This “advantage” was no longer obvious at 4 dpf and 6 dpf (Figures 1(g) and 1(l)); the organization and development of sarcomere in juvenile had no significant difference between the 0.001 mg ATR/L group and the control. In juveniles treated with higher concentrations of ATR (0.01 and 0.1 mg/L), the differentiation of muscle fibers at 2 dpf seemed not obvious, but the density of muscle fibers was slightly lower than the control (Figures 1(c) and 1(d)). Under these two concentrations of ATR, the differentiation of muscle fibers was found at 4 dpf, the boundary between the fast and slow muscle fibers was not clear, and the number of slow twitch fibers was less than the control group's (Figures 1(h) and 1(i)). The abnormal arrangement of the fibers in slow twitch muscle was found clearly at 6 dpf; the slow twitch fibers were arranged to thin-wire types at dorsal section. At the ventral section, the slow twitch was distributed patchily among fast twitch fibers (Figures 1(m) and 1(n)). ATR at the highest concentration (1 mg/L) interfered in the normal development of sarcomere muscle fiber seriously; the density of juvenile sarcomere was lower than the control group's significantly at 2 dpf, and the muscle fibers were arranged loosely (Figure 1(e)). At 4 dpf, though the density of muscle fiber increased normally, there were no clear boundaries between the differentiated muscle fibers, and slow twitch fibers were scarce (Figure 1(j)). At 6 dpf, the development of sarcomere was severely damaged; the boundary between fast and slow twitch could not be observed; and the length of sarcomere was significantly shorter than the control group's (Figure 1(o)). So ATR did not affect the increase in the number of the muscle fibers, but it could make the differentiation and the alignment of fibers abnormal.

Changes of sarcomere length were measured at 6 dpf (Table 1). The length of sarcomere from anterior-posterior axis decreased with the increasing of concentration of ATR. The length of juveniles' sarcomere treated with the very low concentration of ATR (0.0001 mg/L) was 0.240 ± 0.002 mm, which had no significance with the control group whose sarcomere length was 0.254 ± 0.006 mm. The lengths of sarcomere in the groups exposed to lower concentrations of ATR (0.001 and 0.01 mg/L) and high concentration of ATR (0.1 mg/L) were 0.215 ± 0.004, 0.200 ± 0.004, and 0.174 ± 0.002 mm, respectively. In the experimental group exposed to high concentration of ATR (1 mg/L), the length of sarcomere dropped to 0.130 ± 0.043 mm, which was only half of the control group.

The height of sarcomere in the corresponding position of dorsal-ventral axis was used as the body reference; the measured data showed that there was no significant difference between the control and ATR treatment groups (Table 1).

### 3.2. The ATR Inhibits Apoptosis Process of Primary Sensory Neurons at Embryo Stage

The apoptosis widely existed in the early embryonic development of zebrafish. With the tissues and organs being mature, many transitional organs existing in the early development were replaced by terminally differentiated ones which had specific function. This kind of normal physiologic apoptosis process was highly susceptible to the interference of internal and external environmental factors, which led to retain the embryonic cells, tissues, or organs [19].

In order to detect abnormal apoptosis events within the zebrafish nervous system caused by ATR during embryonic development, the experiment analyzed quantitatively the cell death in embryonic neural tube using AO entirely staining method and observed the characteristics distribution of the apoptotic cells. According to the distribution patterns of early apoptotic cells in zebrafish embryos, apoptotic cells began to appear in the head and tail ends from 12 hpf. From 16 hpf to 20 hpf, the number of apoptotic cells increased gradually, they mainly distributed in the middle of the body. Meanwhile, their amounts were slightly more in the tail than in the head. These apoptotic cells were usually cleared by the body within 2 h [17]. So, 20 hpf was chosen as the detection point in this research.

**Figure 2:**
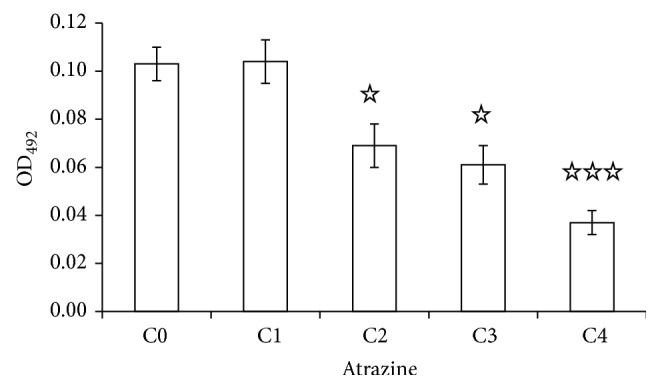
Dose-dependent reduction of cell amount of 20 hpf whole embryos, quantified with AO staining. C0, DMSO; C1, 0.0001 mg/L; C2, 0.001 mg/L; C3, 0.01 mg/L; and C4, 0.1 mg/L. 10 zebrafish embryos were used for each well in 96-well plates and 2 reduplications were taken for each well.

The changes of the number of apoptotic cells in the whole embryo (20 hpf) were calculated (Figure 2). Dead cells within the embryo could absorb AO, so the absorbance value of sample AO to the excitation light (Ex 492 nm) directly reflected its concentration, thus reflecting the number of dead cells in the embryo. There was no significant difference in absorbance value between the very low concentration ATR (C1, 0.0001 mg/L) and the control (C0, DMSO) samples (the value of the control is 0.103; ATR is 0.104). Compared to the control, absorbance value of embryos treated by low concentrations of ATR (C2, 0.001 mg/L, C3 0.01 mg/L) decreased to 0.069 and 0.061, respectively. When the concentration of ATR reached 0.1 mg/L, the absorbance value of embryos dropped to 0.037, which had fallen more than 60%.

**Figure 3:**
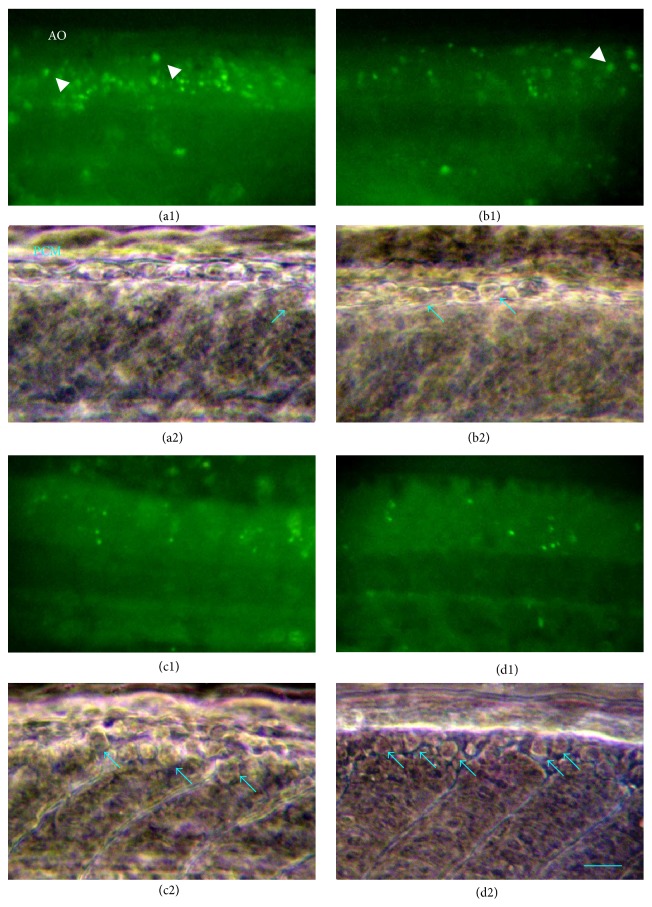
ATR reduced the amount of AO-positive cells in neural tube of 20 hpf embryos, and resulted in the number of RB cells increased in dorsal spinal cord of 72 hpf larvae. Control embryonic neural tube concentrated large apoptotic cells, green colored point is AO^+^ cells, also located in the superficial skin (a1), while showing a small amount RB cells (a2). After treatment of ATR (b 0.001 mg/L, c 0.01 mg/L, d 0.1 mg/L), AO^+^ cells in embryonic neural tube became less (b1, c1, d1); The number of RB neurons increases under the phase contrast microscope (b2, c2, d2). The white arrows indicate the apoptotic RB cells; the blue arrow indicated the survival RB cells. In all photos, juvenile dorsal was to up and the mouth to the right. Scale: (a1, b1, c1, d1) in 100 *μ*m; (a2, b2, c2, d2) of 25 *μ*m. 10 samples were used for each experimental batch. 3–5 batches were reduplicated.

In Figure 3, four fluorescence micrographs of embryos stained by AO showed the apoptotic cells (AO^+^) in embryo of control mainly concentrated upon the back region of the neural tube; the colored spot with larger diameter could be seen in the dorsal edge of the neural tube (Figure 3(a1)). According to the location and size, some small colored dot located in the middle of neural tube might be some intermediary neurons, while the larger colored spot was probably RB neurons. The AO^+^ cells of embryos treated with low concentration of ATR (0.001 mg/L) decreased; a big colored point was still visible in dorsal edge of neural tube (Figure 3(b1)). After the embryos were exposed to low concentration of ATR (0.01 mg/L), the number of AO^+^ cells did not differ from (b1); the big colored dots did not exist in the backside edge (Figure 3(c1)). When the concentration of ATR increased to 0.1 mg/L, the number of AO^+^ cells in neural tube of embryos further reduced; the large colored point was not distributed in the dorsal edge (Figure 3(d1)). The distribution of AO^+^ cells in head and tail of stained embryos did not have characteristic difference. (picture was not shown). The histological evidence was consistent with the above-mentioned quantitative results.

RB neurons were embryonic primary sensory neurons which were the main cell type of AChE expression [17]. During the developmental process, RB neurons were replaced by dorsal root ganglion neurons which had higher transfer efficiency [20]. The apoptosis of RB neurons began at 14 hpf, which peaked at about 60–72 hpf. To confirm the relationship between the reduction of apoptosis of RB neurons and ATR, the number of RB of embryonic neurons was observed* in vivo*. There were a few RB neurons in the control embryos (Figure 3(a2)). With the increase of the concentration of ATR, the number of surviving RB neurons significantly increased (Figures 3(b2), 3(c2), and 3(d2)).

### 3.3. The Variation Trend of AChE Activity Affected by the Increase Concentration of ATR in Embryos and Larvae Was Different

The activity of the AChE within 72 hpf was quantitatively determined in this experiment (Table 2).

In the control group, AChE activity level can be detected in the early embryonic development. At 24 hpf, the values of AChE activity between 0.109–0.128 U were, on average, 0.115 U. AChE activity in the embryos (or in the larvae) gradually increased along with the embryonic development. At 36 hpf, the values were to 0.230 U (0.214–0.232 U); at 48 hpf, rose to 0.554 U (0.537–0.780 U); at 72 hpf, to the highest level, were 3.039 U (2.969–3.075 U). The results of 48 hpf and 72 hpf were consistent with the literature reports [21], so 4–8 dpf AChE activity levels were not continued to measure, and it was deduced by the changes of 72 hpf enzyme activity.

**Figure 4:**
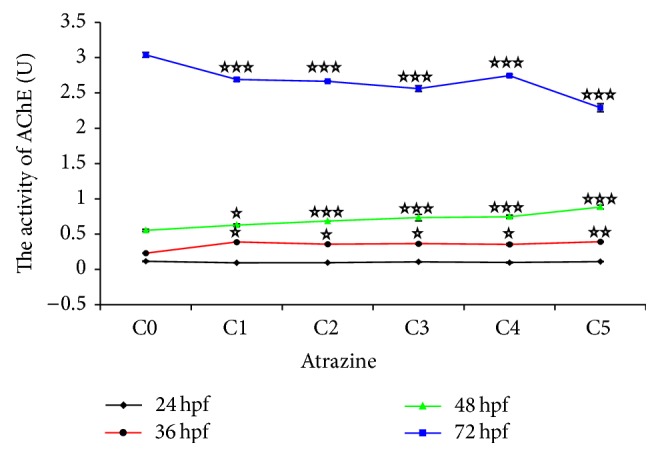
ATR bidirectionally regulated AChE activity of embryos and larvae exposed to ATR at early embryonic stages. This figure was a trend chart made by the data in Table 1. Before hatching (36 and 48 hpf), the high concentration of ATR could increase the activity of AChE; to the contrary, the activity of AChE was inhibited by the high concentration of ATR after incubation. Figure in the ATR concentration gradient (mg/L): C0, 0 (DMSO); C1, 0.0001; C2, 0.001; C3, 0.01; C4, 0.1; and C5, 1. 30 zebrafish embryos were used for each experimental group (24 hpf, 36 hpf, 48 hpf, and 72 hpf).

Relationship between AChE activity and the concentration of the ATR was mapped according to the results of measurement (Figure 4). The AChE activity showed a rising trend with the increasing concentration of ATR before hatching. At 24 hpf, the activity of enzyme in test groups and control group remained in basal level. There was no obvious difference between them. At 36 hpf the activity of enzyme in all groups dealt with ATR was higher than the control's, which increased significantly. The curve of activity of enzyme at 48 hpf showed a slightly rising trend; the activity rose with the increase of concentration of ATR. After hatching (72 hpf), the curve of AChE activity showed downward trend with the increase of concentration of ATR; the inhibition to all the groups treated with ATR was significant; enzyme activity had the greatest reduction with 1 mg ATR/L (25%).

**Figure 5:**
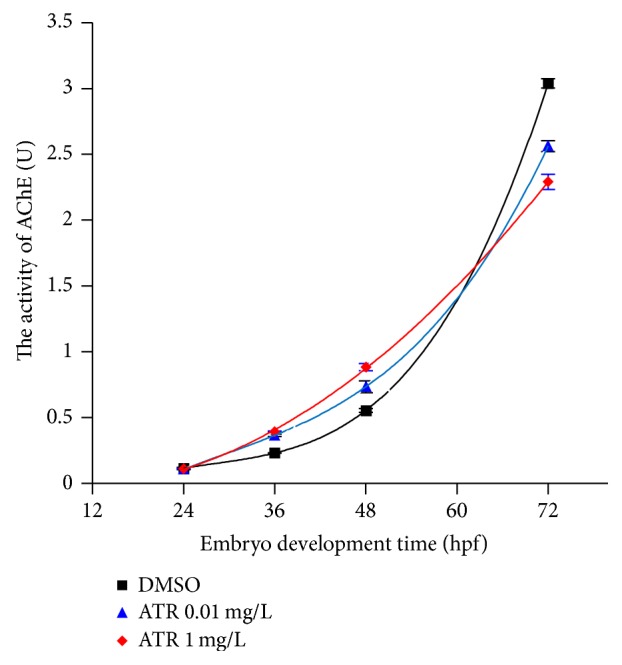
Exposure under ATR slowed down developmental increase of AChE activity. The exponential fit of the AChE activity dealt with different concentrations of ATR. DMSO was the solvent control; 0.01 mg/L and 1 mg/L, respectively, represent the trend of low concentration and high concentration of ATR. Curve was becoming flat from steep with the increasing of the ATR concentration. 30 zebrafish embryos were used for each experimental group (24 hpf, 36 hpf, 48 hpf, and 72 hpf).

Fitted curve of AChE activity over time was described according to the formula: *Y* = *A*_1_exp⁡(*X*/*t*_1_) + *Y*_0_. The activity of AChE during the zebrafish embryonic development presented typical exponential growth. With the increase of the concentration of ATR, the curve became flat (Figure 5).

## 4. Discussion

### 4.1. ATR Inhibited Spontaneous Neural Activity, Leading to the Reduction of Activity-Dependent Apoptosis of RB Neurons

Spontaneous activity of neurons was the earliest electrical activity and had multiple physiological functions in the early development. The pattern of embryo spontaneous activity was different from the adult's; its characteristic was decided by receptors or channels expressed in membrane surface during embryonic period. With the body being mature, the effect of spontaneous activity on downstream was changing the numbers and characteristics of receptors and channels expressing on the membrane surface, resulting in the change of pattern of neural activity [22, 23]. A lot of researches confirmed that electrical activity and potential depolarization could protect the synaptic connection which was established in the embryo and participated in the neural activity of neurons and promoted its maturity by selecting mechanism [24, 25]. But neurons' surviving in development was not always derived by the activity of neurons. During the embryo development of zebrafish and* Xenopus laevis*, their RB neurons and Cajal-Retzius neurons of mammalian existed briefly in the early development and then entered the programmed cell death with the nerve activity being mature. The excitotoxic effect of neural activity can be initiated by the rising of NMDA receptor expression levels in development, and then the NMDA receptor formation unremitting inward calcium electricity repeatedly stimulated action potential which was mediated by sodium current [26–28]. The blocking of the Na^+^ current mediated action potential would reduce the death of RB neurons of zebrafish; this phenomenon can be detected by gene manipulation (mutant strain established by gene knock out) and pharmacology blocking [29]. It was speculated that the reduction of the apoptosis of RB neurons induced by ATR may be due to the lack of the action potential. In other words, spontaneous neuronal activity in embryonic stage or larval stage could be interfered by ATR.

Spontaneous neuronal activity played an important role in the building of the embryonic neurons. It was not only necessary to maintain the synaptic connection, but also helpful for consolidating the functions of the synapse via inducing the activity-dependent expression of the gene. With electrical coupling between embryonic cells disappearing, spontaneous calcium potential depending on the inactivation of voltage appeared, inducing sharply depolarization spontaneous sodium potential [30]. In the RB neurons of zebrafish, two subtypes of voltage-gated sodium channels were mainly expressed: Nav1.1 and Nav1.6 [31, 32]. Voltage-gated sodium channels of Nav1.1 mediated RB neurons forming sodium current in the early development. However, the number of Nav1.6 increased with the development process. The increase of the expression of Nav1.6 was necessary for the upregulation of sodium current of the action potential [33, 34]. Antisense nucleic acid was injected to the zebrafish embryo to block the function of Nav1.6, and then its RB cells apoptosis was inhibited, and subprime primary motor neurons and motor neurons innervation of muscle tissue decreased at the same time [35]. These phenomena were very similar to the experimental observations. So Nav1.6 may be the target of the ATR.

### 4.2. ATR Disturbed the Construction of Neural Circuits in Zebrafish

As the other lower vertebrates, the process of zebrafish neurogenesis had two climaxes [36]. Initial neurogenesis started at the later phase of the gastrulae and lasted the whole process of embryogenesis, resulting in large primary neurons, such as M cells, RB neurons, and three-class primary motor neurons (CaP, MiP, and RoP). The neural structures of embryo and juvenile fish were made of these primary neurons. The second neurogenesis appeared in postembryonic period (2 dpf), producing more precise and complex neural networks instead of the original ones [37]. The complication of neural networks did not involve the replacement of neurons structure but involved the axonogenesis and the establishment of functional synaptic connections among large number of neurons [38].

ATR had the potential to disrupt the neuroendocrine activity and affected the normal function of dopaminergic neurons [8]. So it was likely to function in the above neurogenesis sensitive period. The conversion from embryonic primary sensory neurons to mature primary sensory neurons existed in the development process of zebrafish motor control center where RB neurons were replaced by dorsal root neurons (DRG). The apoptosis of RB neurons disappeared completely in 5 dpf under normal circumstances. At this time, the numbers of DRG neurons showed an upward trend. In 5 dpf, they became the dominated neurons to dominate the movement of trunk. In the alternate process, the apoptosis of RB neurons was necessary. The study of* macho* (*mao*) mutant showed that the reduction of RB neuronal apoptosis would inhibit DRG neurons projecting outwards [39]. When zebrafish embryos or juvenile were exposed to ATR environment, the apoptosis of RB neurons did not happen normally. It was inferred that ATR was likely to impact the process of second neurogenesis and the building of the advanced neurological function after hatching. The level of AChE activity did not fluctuate with the increase of the concentration of ATR. It was indicated that the influence for first neurogenesis caused by ATR did not reflect the change of the AChE activity; but the fact that ATR had no effect on neurogenesis at this stage was not confirmed.

### 4.3. AChE Was Not Directly Targeting for ATR

AChE played an important role in the development process of nervous system, except the classic activity; it also had nonclassical activity such as neurotrophic activity. In most toxicology and pharmacology experiments, the abnormality of motor function appeared; the AChE activity often acted as the molecular marker or the basis of explanation [40]. AChE appeared earlier than the acetylcholine during the process of development and existed in many nonneural tissues, so it also had many important biological classic functions, such as induction of the occurrence of neurite and the activity of neurotrophic factors and promoting the adhesion of nerve cells. Inhibition of non-classic activity of AChE was harmful to the normal development and differentiation of muscle fiber that induced by nervous system. The* ache* mutant strains of zebrafish showed sarcomere dysplasia, innervation area becoming narrow, slow response to mechanical stimulation, and so forth [41]. These phenomena were similar with experimental results in this research; but this article did not have enough evidences to demonstrate that AChE activity can be inhibited by the ATR directly. So the effects of ATR on AChE may occur in* ache* gene transcriptional level or in the posttranscriptional level.

In the report about the effects of chlorpyrifos (CPF) on the activity of AChE, the value and the changing trend of enzyme activity in the developmental process of zebrafish from the control group were similar to this experiment's. The value of enzyme activity was stable at about 35 U after 6 dpf. However, the AChE activity of the test groups (10 ng CPF/mL and 100 ng CPF/mL) was continuously lower than the control's [42]. This can prove that the ATR had a toxicology effect in a way which was different from the pesticides (such as CPF) that only inhibited AChE activity.

The apoptosis of RB neurons would be inhibited by ATR, which was different from the phenomenon that the number of RB apoptosis increased in the* ache* mutant. In* ache* point mutations strain of zebrafish, the AChE activity of the hydrolysis of acetylcholine was knocked out, but their normal expression was not suppressed. The defects of formation and innervation of muscle fibers were found in mutant embryos, and RB neurons apoptosis occurred untimely. The loss-of-function experiments of nAChR *α* subunit mutation strain showed that the damage in neuromuscular development was mediated by the nAChR in* ache* mutation strains, not due to the decline of neuronal activity. So, AChE was not the sufficient condition in the behavioral effects mediated by ATR. In addition, the AChE had a broad effect on the swimming act of damaging zebrafish larvae caused by ATR, which demonstrated that AChE may be in the downstream of the ATR pathways, causing abnormal physiological phenomena.

## 5. Conclusions

In the embryos exposed to ATR, the fast twitch fibers dysplasia and abnormal sarcomere were found. After hatching, AChE activity affected by ATR reduced which reflected that the innervation of the trunk sarcomere was abnormal. In the embryos treated with ATR, embryonic primary sensory neurons RB cells couldn't enter the normal apoptosis program; it would interfere with the mature process of sensory neurons and result in the feeling function abnormality in the larvae.

## Figures and Tables

**Table 1:** Length of trunk myotomes of larvae (6 dpf) ATR-treated at early development.

ATR (mg/L)	Length			Height		
	(mm)	*n*	Sig	(mm)	*n*	Sig
0 (DMSO)	0.254 ± 0.006	16	No	0.410 ± 0.007	16	No
0.0001	0.240 ± 0.002	17		0.404 ± 0.005	17	
0.001	0.215 ± 0.004	16	☆	0.393 ± 0.003	16	
0.01	0.200 ± 0.004	20		0.395 ± 0.006	20	
0.1	0.174 ± 0.002	20		0.384 ± 0.004	20	
1	0.130 ± 0.004	20	☆☆☆	0.390 ± 0.059	20	

Note: *n*: the number of the samples; Sig: significant difference.

**Table 2:** AChE activity of embryos and larvae treated with ATR at early development.

ATR (mg/L)	24 hpf			36 hpf			48 hpf			72 hpf		
	AChE (U)	*n*	Sig	AChE (U)	*n*	Sig	AChE (U)	*n*	Sig	AChE (U)	*n*	Sig
0 (DMSO)	0.115 ± 0.007	3	—	0.230 ± 0.002	3	—	0.553 ± 0.015	3	—	3.039 ± 0.035	3	—
0.0001	0.094 ± 0.002	3	No	0.388 ± 0.004	3	☆	0.629 ± 0.016	3	☆	2.690 ± 0.026	3	☆☆☆
0.001	0.097 ± 0.002	3		0.359 ± 0.006	3		0.685 ± 0.001	3	☆☆☆	2.666 ± 0.008	3	
0.01	0.107 ± 0.003	3		0.365 ± 0.009	3		0.734 ± 0.046	3		2.562 ± 0.042	3	
0.1	0.097 ± 0.007	3		0.356 ± 0.013	3		0.747 ± 0.022	3		2.745 ± 0.019	3	
1	0.112 ± 0.002	3		0.393 ± 0.005	3	☆☆	0.883 ± 0.027	3		2.290 ± 0.058	3	

Data are expressed as mean ± standard error of mean. *n*: the experimental batches; Sig: significant difference.
